# Short and long-term phytoremediation capacity of aquatic plants in Cu-polluted environments

**DOI:** 10.1016/j.heliyon.2023.e12805

**Published:** 2023-01-10

**Authors:** Brendan Enochs, George Meindl, Grascen Shidemantle, Vanessa Wuerthner, David Akerele, Allison Bartholomew, Benjamin Bulgrien, Abigail Davis, Katelynn Hoyt, Lena Kung, Maria Molina, Elias Miller, Ally Winship, Yiqun Zhang, Joseph Graney, David Collins, Jessica Hua

**Affiliations:** a Environmental Studies Program, Binghamton University, Binghamton, NY, USA; b Department of Forest and Wildlife Ecology, University of Wisconsin-Madison, Madison, WI, USA

**Keywords:** Phytoremediation, Copper, Freeze thaw cycles, Physa acuta, Lemna minor, Vallisneria americana

## Abstract

Freshwater ecosystems face numerous threats from human populations, including heavy metal contamination. Phytoremediation, the use of plants to remediate contaminated soils and sediments, is an effective and low-cost means of removing chemical contaminants, including heavy metals, from polluted environments. However, key questions remain unanswered in the application of this technology in aquatic environments, such as the long-term fate of pollutants following plant uptake. In this study, using two common wetland plant species (duckweed and tape grass), we first examined the capacity of plants to remove copper (Cu) from polluted water. Next, we evaluated the leaching potential of plant tissues following decomposition and how it is affected by a simulated freeze-thaw cycle. Using phytoremediated water and leachates from senesced plants we assessed phytoremediation success and Cu leaching potential by conducting standard toxicity assays using pond snails (*Physa acuta*), a species with known Cu sensitivity. We found that duckweed outperformed tape grass as a phytoremediator at low Cu concentrations. In addition, for plants grown in low concentrations of Cu, leaching from decaying plant material did not negatively impact snail survival, while at high concentrations of Cu, leaching did result in toxicity. Lastly, we found that a simulated freeze-thaw cycle increased the release of Cu from plant tissue in the presence of high Cu concentrations only, resulting in reduced snail survival. Our results indicate that in moderately Cu-polluted environments, some aquatic plants can remove contaminants without a long-term risk of leaching. In contrast, phytoremediation in highly polluted environments will likely require removal of plant tissue to prevent leaching of previously accumulated metals. Land managers must not only consider plant species and degree of contamination, but also geographic location, as freeze-thaw cycles may enhance plant decomposition and increase the likelihood of contaminant leaching following phytoremediation efforts in aquatic ecosystems.

## 1 Introduction

The growth of the human population and associated increases in resource use and pollution are regarded as leading threats to biodiversity and ecosystem health globally [1]. Freshwater ecosystems are especially susceptible to human impacts given the tendency of humans to develop and inhabit areas alongside freshwater environments [2]. Freshwater habitats, occupying less than 1% of the Earth's surface area, support disproportionately high levels of biodiversity [3] and provide vital life-supporting ecosystem services to human populations [4]. Despite their importance, freshwater ecosystems face many anthropogenic threats, including water extraction [5], invasive species [6], climate change [3], and chemical pollution [7].

The introduction and persistence of chemical pollutants, including heavy metals, can have lasting negative impacts in freshwater environments [8]. In particular, the heavy metal copper (Cu) has been concentrated in soils, sediments, and water by human activities [9]. While Cu is an essential nutrient for many organisms due to its integral function in enzyme activity [10,11], organisms often experience toxicity when exposed to concentrations of Cu that exceed physiological requirements [12,13]. Copper sulfate (CuSO_4_) is a common source of Cu pollution in the environment, especially in agricultural settings where it is used as a biocide or fertilizer supplement [14]. Copper sulfate is often utilized in the control of unwanted species, with approximately 9–11 million pounds of elemental Cu applied annually in the U.S. to control algal blooms [15]. Anthropogenic sources of Cu are often transported to freshwater systems by way of surface runoff or direct application, where it accumulates and results in toxicity to non-target organisms [16]. Freshwater species are particularly vulnerable to Cu pollution, which is lethal at low environmental concentrations in benthic invertebrates, fish, amphibians, and zooplankton [17,18]. More specifically, Cu exposure can cause direct mortality in a variety of organisms, including mollusks [19], decrease the reproductive success of copepods [20], induce behavioral changes and stunted growth in larval amphibians [21], and alter community structure and trophic interactions [22]. Understanding the effects of Cu contamination and exploring ways to advance mitigation efforts are essential for managing human impacts on freshwater ecosystems in human dominated landscapes.

Many techniques for the removal of heavy metals have been developed and extensively studied, including chemical precipitation, absorption, membrane filtration, coagulation-flocculation, flotation, and ion exchange [23]. However, these methods are often limited due to their cost and disposal methods, which prevents their use at a large scale and may be problematic when employed in natural environments [24]. Phytoremediation, the use of plants and their associated microbes to enhance the biodegradation and removal of pollutants, has emerged as a tool to assist in the decontamination of aquatic environments [25,26]. Some effective phytoremediators are well adapted to tolerate metal toxicity and can sequester large amounts of metal contaminants [25,27]. Phytoremediation can be remarkably efficient as some plants, termed metal hyperaccumulators, can accumulate metals well over 1% of their dry weight, which is orders of magnitude higher than most plants [28]. Phytoremediation is also considered a cost-effective and environmentally friendly alternative to conventional removal strategies [29].

Although there are apparent advantages of phytoremediation in the removal of heavy metal pollutants in aquatic environments, the strategy still has constraints that must be addressed. Specifically, phytoremediation is only a temporary removal mechanism, and it usually requires harvest and disposal of plant material following metal sequestration; otherwise, the plants may become another source of pollutants during decomposition [29]. Generally, aquatic plants used for phytoremediation are harvested and then repurposed as biofuels, composted, or disposed through incineration [30]. However, these methods may not be applicable in all situations (e.g., financial limitations or risks of directly impacting ecosystems). Additionally, changes in water movement may displace plant material before harvesting can take place [31]. Therefore, in order to improve the efficacy of phytoremediation using aquatic plants, it is imperative to better understand the fate of heavy metals following sequestration and their potential for leaching. A possible confounding factor on the fate of sequestered heavy metals is the influence of freeze/thaw cycles (FTCs) during the decomposition process. For example, decomposition and nutrient release from terrestrial plant litter are stimulated by freeze/thaw cycles (e.g. Refs. [[32], [33], [34]]). However, little is known about the effect of FTCs on the decomposition of aquatic plants or their ability to influence the release of accumulated metals from plants used to remediate contaminated freshwater environments. This information is critical for management efforts to be successful in temperate climates worldwide.

In this study, we tested the phytoremediation potential of the aquatic plant species *Lemna minor* and *Vallisneria americana,* and whether these plants could reduce Cu concentrations below toxicity thresholds for freshwater snails. In accordance with studies that have demonstrated the potential of *L. minor* to accumulate copper [[35], [36], [37], [38]] and possibility of *V. americana* to perform as a bioindicator of Cu in the environment [39], we predicted that both plants would effectively remove Cu, especially at low concentrations. Following Cu sequestration, we also sought to determine whether plants leach Cu back into the environment following death, and whether leaching potential is affected by FTCs. Although leaching from plant material is often regarded as a concern with aquatic phytoremediation strategies [29], we hypothesized that leaching may be negligible in systems with low Cu concentrations. On the other hand, we predicted that plant tissues exposed to FTCs would increase leaching of Cu regardless of concentration.

## 2 Materials and methods

### 2.1 Study system

We utilized two common macrophytes for this study: *Vallisneria americana* (Hydrocharitaceae; hereafter “tape grass”) and *Lemna minor* (Lemnaceae; hereafter “duckweed”). Tape grass and duckweed are both widely distributed across the continental United States [40], and therefore make suitable candidates for large-scale use as phytoremediators in contaminated water bodies. Specifically, tape grass has been demonstrated to be an effective bioindicator of sediment phytotoxicity in contaminated aquatic systems [41]. Members of the *Vallisneria* genus, notably *V. spiralis*, have shown the ability to sequester high concentrations of heavy metals such as Cu [42], in addition to being competent phytoremediators of a wide array of contaminants [[43], [44], [45]]. Similarly, duckweed, along with other members of the Lemnaceae, have long been utilized in the removal of unwanted pollutants and is known to bioremediate wastewater contaminated with Cu and other heavy metals [37]. All plants utilized here were obtained from the aquarium plant wholesaler aquariumplants.org (Florida, USA).

To understand the effectiveness of tape grass and duckweed in decontaminating Cu-polluted aquatic systems, we used a common freshwater gastropod as a bioindicator, *Physa acuta*. *Physa acuta* is regarded as one of the most widespread gastropods across North America and the world, with populations in Europe, Asia, Australia, and South Africa [46,47]. Previous work by Gao et al. [48] demonstrated that *P. acuta* has a strong sensitivity to Cu, thus making *P. acuta* an ideal model to assess the success of phytoremediation efforts in our study. We collected all *P. acuta* (hereafter “snails”) from wild populations at the Apalachin Marsh Trail in Apalachin, NY, USA (42°05′21.0″N 76°10′17.1″W). Before use, we acclimated snails within bins containing 4 L of UV-treated filtered well water (100 snails per bin) under a photoperiod regime of 12 h light: 12 h dark, at a constant temperature of 24 °C and fed a spirulina mix *ad libitum*.

We generated experimental microcosms (five-gallon buckets; n = 72) on February 7, 2019, each with 2 L of Mystic White® II pure quartz sand (SiO_-----2_) and 3 gallons of UV-treated filtered well water. We added filtered well-water every week in order to maintain volume. On February 14, 2019, we added: 1.5 g of tape grass (planted), 1.5 g of duckweed (floating on water surface), or no plant matter to the microcosms to create three plant treatments (N = 24 buckets per treatment). We maintained buckets in the Binghamton University research greenhouse facility. We supplemented buckets with Seachem® fertilizer, including nitrogen, phosphorous, and potassium, every 2 weeks.

### 2.2 Experiment 1: evaluating phytoremediation capacity

After a 10-day plant acclimation period (February 23, 2019), we added Cu to the buckets to create a low Cu treatment (1 ppm of Cu -added as CuSO_4_5H_2_O, Thermo Fischer Scientific, Catalog #197730250) and a high Cu treatment (10 ppm of Cu). We chose these concentrations of Cu because they are consistent with concentrations implemented in previous studies and reflect realistic levels of contamination [49]. Specifically, copper sulfate is directly applied to freshwater bodies as an algaecide at species-dependent concentrations ranging from 0.25 to 2 ppm [15], and Cu concentrations in wastewater effluent may exceed 10 ppm [14]. Buckets were arranged in a randomized block design consisting of 8 blocks. Each of the treatment combinations of Cu (0, 1, or 10 ppm) and plant species (tape grass, duckweed, or no plant) were assigned to every block, to help account for any heterogeneity in the distribution of environmental conditions within the greenhouse. To confirm that the water quality within bucket microcosms did not significantly vary across treatments, we measured dissolved oxygen, pH, phycocyanin concentration, and chlorophyll *a* concentration using an EXO Sonde (YSI) five weeks after Cu addition and found no differences across plant species or Cu treatments.

To determine the phytoremediation capacity of tape grass and duckweed, at three time points (immediately after Cu treatment, four weeks after treatment, and eight weeks after treatment), we collected water samples from the buckets to be used in snail time to death (TTD) assays. In the TTD assays, snails were individually exposed to 10 ml water samples (N = 10 snails per bucket) at every time point and mortality was recorded every 4 h for a total of 96 h. We considered snails dead if no movement occurred for 30 s after water was swirled in their containers. Following completion of the TTD assays, we weighed and preserved snails and stored water samples for later chemical analysis to confirm relative differences in Cu concentration across treatments (see Appendix 1). Plant specimens of tape grass and duckweed from all treatment buckets were collected, rinsed, air dried, and then stored in a humidity-controlled environment for use in Experiment 2.

### 2.3 Experiment 2: evaluating Cu toxicity due to leaching from senesced tape grass and duckweed

#### 2.3.1 Leaching

To create leachate solutions, we used leaf tissue samples of tape grass and duckweed collected under different Cu concentrations (0, 1, 10 ppm) from Experiment 1. On February 4, 2020, we combined 2 g of leaf tissue and 200 ml of UV-treated filtered well water for each treatment. We created control solutions with 200 ml UV-treated filtered well water and no plant material. On April 6th, 2020, we conducted TTD assays (N = 15 snails per treatment) following the same procedure as Experiment 1. Water from each treatment was collected and analyzed to confirm differences in Cu concentration across treatments (see Appendix 1).

#### 2.3.2 Leaching after freeze-thaw cycles

To simulate leachate solutions that experience freeze thaw cycles, we used leaf tissue samples of duckweed collected under different Cu concentrations (0, 1, 10 ppm) from Experiment 1. Duckweed was solely used for the freeze/thaw cycle treatments because of inadequate remaining biomass for tape grass. To create leachate solutions, on February 4, 2020, we collected 2 g of duckweed tissue from each of the Experiment 1 Cu treatments (0, 1, and 10 ppm) and added it to 200 ml of UV-treated filtered well water. We also generated a no duckweed control (200 ml of UV-treated filtered well water). We then simulated a freeze/thaw cycle by placing all solutions into a −4 C freezer on February 18, 2020. After one week, we removed solutions from the freezer and held solutions at room temperature (20 C). After one week at room temperature, we placed solutions back into the freezer for one more week. We added water weekly to the leachate solutions to maintain volume. On April 6th, 2020, using the solutions that underwent the freeze-thaw cycle, we conducted TTD assays (N = 15 snails per treatment) following the same procedure as Experiment 1. Water from each treatment was collected and analyzed to confirm differences in Cu concentration across treatments (see Appendix 1).

### 2.4 Statistical analyses

#### 2.4.1 Experiment 1: evaluating phytoremediation capacity

We conducted Kaplan Meier Survival analyses with a Breslow (Generalized Wilcoxon) test to determine if there were differences in the survival distribution of snails exposed to the three plant treatments. We conducted a separate analysis for each of the Cu treatments and at each timepoint. For significant Breslow omnibus tests, we ran Breslow pairwise comparisons to determine which plant leachate treatments resulted in different snail survival distributions. A Bonferroni correction was made with statistical significance accepted at the *p* < 0.016 level.

#### 2.4.2 Experiment 2: evaluating Cu toxicity due to leaching from senesced tape grass and duckweed

We conducted Kaplan Meier Survival analyses with a Breslow (Generalized Wilcoxon) test to determine if there were differences in the survival distribution of snails exposed to the plant leachate treatments. For significant Breslow omnibus tests, we ran Breslow pairwise comparisons to determine which plant leachate treatments resulted in different snail survival distributions. A Bonferroni correction was made with statistical significance accepted at the *p* < 0.0125 level.

Additionally, to confirm that snails in the time to death assay in the different plant leachate treatments did not significantly differ in size, we conducted an analysis of variance (ANOVA). For snails exposed to plant leachates from the 2019 1 ppm copper treated mesocosms, we found a significant omnibus effect of treatment (F_10, 145_ = 1.9; p = 0.04). However, none of the pairwise comparisons between treatments were significant (*p* > 0.21).

## 3 Results

### 3.1 Experiment 1: evaluating phytoremediation capacity

**Fig. 1:**
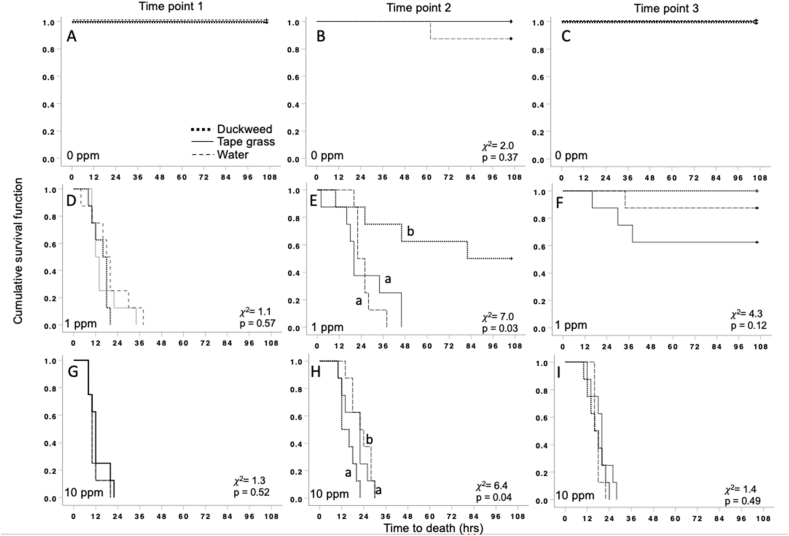
Differences in survival distributions of snails in the no plant, tape grass, and duckweed mesocosms at three time points with 0 ppm Cu added (A–C), 1 ppm Cu added (D–F) and 10 ppm Cu added (G–I). Time point 1 was immediately after Cu treatment, time point 2 was four weeks after treatment, and time point 3 was eight weeks after treatment.

The survival distributions were not significantly different for snails exposed at time points 1 and 3 (Fig. 1; p > 0.00.05). In contrast, at time point 2, the survival distributions for snails exposed to 1 ppm and 10 ppm Cu were significantly different (Fig. 1E and H; χ^2^(2) = 7.0, p = 0.03 and χ^2^(2) = 6.4, p = 0.04, respectively).

For snails exposed to 1 ppm of Cu, there was a statistically significant difference in survival distributions for snails in the duckweed treatment compared to both the tape grass and water treatments (p < 0.016). The survival distribution of snails from the tape grass treatment did not significantly differ from snails in the water treatment.

For snails exposed to 10 ppm of Cu, there was a statistically significant difference in survival distributions for snails in the water treatment compared to both the tape grass and duckweed treatments (p < 0.016). The survival distribution of snails from the tape grass treatment did not significantly differ from snails in the duckweed treatment.

### 3.2 Experiment 2: evaluating Cu toxicity due to leaching from senesced tape grass and duckweed

**Fig. 2:**
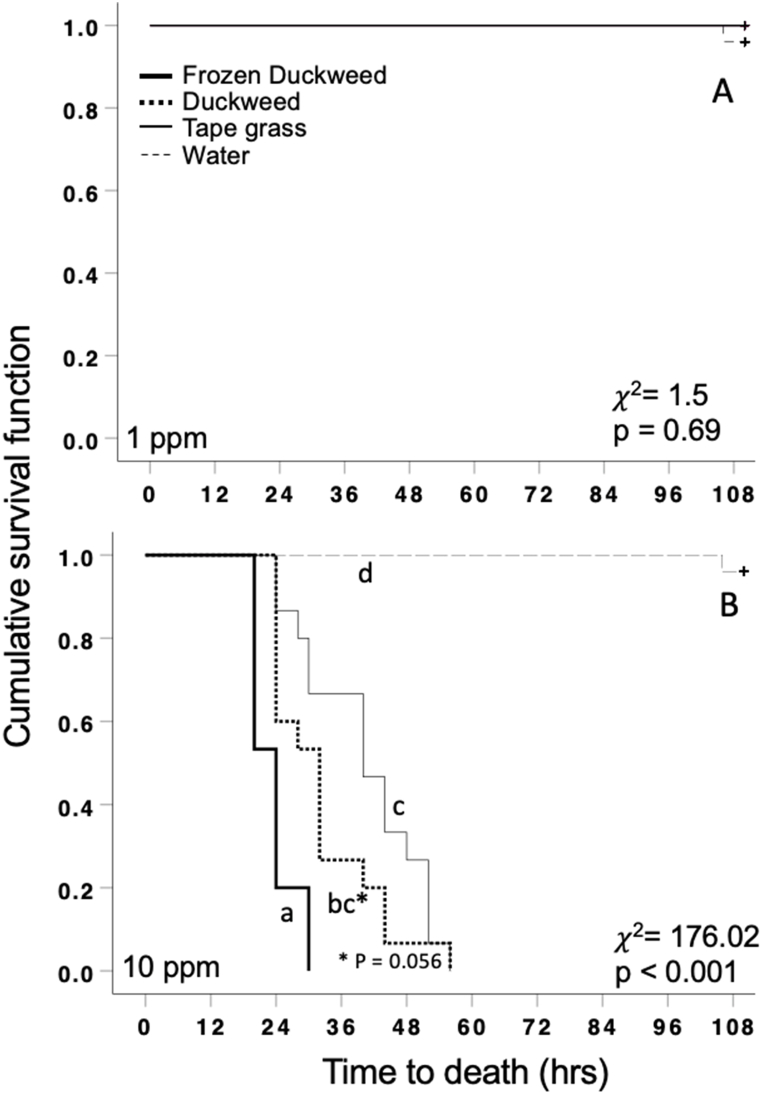
Differences in survival distributions of snails exposed to the four plant leachate treatments from the (A) 2019 1 ppm Cu treated mesocosms and (B) 2019 10 ppm Cu treated mesocosms.

We conducted a separate analysis for plant leachates collected from the 2019 1 ppm vs. 2019 10 ppm mesocosms. The survival distributions were not significantly different for snails exposed to plant leachates from the 2019 1 ppm mesocosms (Fig. 2A; χ^2^(3) = 1.5, p = 0.69). In contrast, the survival distributions were statistically significantly different for snails exposed to the different plant leachates from the 2019 10 ppm mesocosms (Fig. 2B; χ^2^(3) = 176.02, p < 0.001). There was a statistically significant difference in survival distributions for the control and frozen duckweed treatment compared to all other treatments (p < 0.0125). The survival distributions for snails exposed to non-frozen duckweed and tape grass did not significantly differ (p = 0.056).

## 4 Discussion

Aquatic ecosystems are threatened by numerous anthropogenic activities that lead to the introduction and persistence of chemical pollutants, including heavy metals such as Cu. Phytoremediation, the utilization of plants in the removal of unwanted environmental pollutants, has been effectively used to remove Cu from aquatic ecosystems (e.g., see Ref. [37]). In this study, we explored the potential of duckweed and tape grass to ameliorate Cu toxicity for freshwater aquatic ecosystems. Furthermore, we tested whether plants leach Cu back into the environment following sequestration and decomposition, and whether release is affected by annual FTCs. We found that duckweed was the only adequate phytoremediator at low Cu concentrations (1 ppm), but neither duckweed nor tape grass were effective remediators at high Cu concentrations (10 ppm). Additionally, snails exposed to leachate solutions of duckweed and tape grass used to phytoremediate Cu at low concentrations (1 ppm) had a low mortality rate that was similar to snails exposed to control conditions. In contrast, snails exposed to leachate solutions from duckweed and tape grass used to phytoremediate high concentrations of Cu (10 ppm) experienced high mortality at a rate significantly faster than snails exposed to control conditions. Furthermore, we found support that annual FTCs may enhance leaching of Cu from plant tissues back into aquatic environments in systems polluted with high concentrations of Cu (10 ppm). Our results highlight the potential of phytoremediation to remove toxic heavy metals from freshwater environments, but also indicate certain environmental features (i.e., plant species, level of Cu pollution, environmental conditions) may dictate the ultimate success of such remediation strategies.

Our two focal plant species, duckweed and tape grass, varied in their abilities to remediate low levels of Cu pollution (1 ppm) in aquatic environments. We found that snails exposed to water contaminated with low concentrations of Cu survived better when duckweed was present compared to contaminated water where there were no plants present or contaminated water with tape grass present. Our finding that duckweed can successfully phytoremediate Cu is consistent with previous work [35,37,50,51]. Indeed, duckweed is commonly utilized in the remediation of chemical pollutants from aquatic environments [38], and more specifically has demonstrated exceptional success in removing heavy metals such as Cu, especially at lower concentrations [35–37]. In contrast, and despite previous work with *Vallisneria* spp. demonstrating success in the phytoremediation of unwanted pollutants [43–45], our data suggests tape grass is a less suitable candidate for phytoremediation of Cu. Specifically, we found that snails exposed to water contaminated with 1 ppm of Cu survived similarly regardless of whether tape grass was present or not. The growth habits of tape grass and duckweed, as well as the experimental design, may help account for the differential success in removing Cu in this experiment. Specifically, due to the free-floating nature of duckweed, its roots remain suspended in the water column giving them direct access to uptake metals either passively or through active transport into the plant body [26]. Due to the relatively short duration of our study, and the use of a sandy substrate rather than finer sediment, it was likely that the concentration of Cu was higher in the water compared to the rooting substrate, making Cu readily accessible to duckweed but not tape grass. Submerged macrophytes, such as tape grass, often outperform their free-floating counterparts in accumulating heavy metals because a major reservoir of metals in natural environments lies within the sediment [52]. Interestingly, we found that at the high concentration of Cu, snails exposed to Cu contaminated water survived longer when both tape grass and duckweed were present compared to when there was just water. However, regardless of the plant treatment, we found 100% snail mortality suggesting that this significant difference in mortality rate is likely not biologically relevant. Future work utilizing finer substrates and longer-term metal exposure will provide additional insights to aid land managers in selecting suitable species for use in phytoremediation across different habitats.

Differences in snail survival across treatments were also evident across sampling times. Specifically, for both low and high Cu treatments, we found that immediately after Cu addition (time point one), snails had similar mortality rates regardless of plant treatment. However, at time point two, snails exposed to the duckweed 1 ppm treatment had longer survival rates compared to tape grass and no plant treatments suggesting that Cu phytoremediation by duckweed can occur as quickly as four weeks following a contamination event. At time point three (eight weeks), overall snail survival was higher compared to survival at four weeks. While natural quartz sand has a low sorption capacity due to low surface area and few sorption sites and is not known to bind high concentrations of metals, including Cu [53], over time, some Cu likely became bound to quartz sand particles used as a substrate in our experiment. Thus, one potential explanation for why mortality is lower across all treatments at time point three is that it took eight weeks for Cu to enter the substrate. Entering the substrate may contribute to lower mortality in the control and tape grass treatments by removing Cu from the water column where we collected water for the snail TTD assay. Additionally, submerged macrophytes most effectively remove contaminants from sediments [52], thus it is possible that it takes until time point three for Cu to be available for tape grass to effectively phytoremediate. Future studies evaluating and measuring Cu concentration in plant tissue are necessary to parse apart the fate of Cu in the presence of tape grass. Collectively, rapid removal of Cu following contamination events is critical to protecting wildlife populations. Towards this goal, our results suggest that for most rapid removal of Cu, duckweed was the most effective.

The rerelease of chemical pollutants following plant death is often a concern in phytoremediation approaches [29]. Historically, such concerns have been remedied by harvesting plant material following pollutant extraction and either disposing or repurposing the contaminated byproduct [30]. Here, we found snails placed in leachate solutions of duckweed plants exposed to low concentrations of Cu (1 ppm) survived the full 96 h observation period. Duckweed is an excellent phytoremediator due to high growth rate, the ability to grow in nutrient poor environments, and tolerance to pH and temperature variation (reviewed in Ref. [54]). In addition, dry biomass of duckweed has strong adsorption capacity to bind metals even following plant death/senescence [55], potentially reducing or slowing leaching of metals back into the environment following plant uptake. This feature of duckweed may reduce the need to remove plant biomass after sequestration in moderately polluted environments, which would otherwise be an economic constraint in phytoremediation implementation in aquatic habitats. Leachate solutions from tape grass exposed to low concentrations of Cu (1 ppm) also resulted in high snail survival. However, in the initial phytoremediation experiment, tape grass did not lower TTD of snails relative to controls at 1 ppm of Cu (unlike duckweed). Therefore, it is likely that the tape grass tissue utilized contained less Cu, and subsequently had a lower capacity for leaching.

Leachates from duckweed and tape grass exposed to high concentrations of Cu (10 ppm) were both toxic to snails (Fig. 2B). Therefore, the phytoremediation of water bodies polluted with high concentrations of Cu may require the removal of plant tissues, regardless of plant species utilized, before decomposition. While both leachates resulted in relatively fast TTD in pond snails, duckweed leachates were marginally more toxic to snails compared to tape grass leachates (P = 0.056; Fig. 2B). Variation in Cu leaching between duckweed and tape grass may be mediated by a number of factors. For example, the chemical compositions of species can result in varying rates of decomposition, as decomposition can vary in accordance with differing concentrations of carbon, phosphorus, and nitrogen within plant tissues [56,57] Alternatively, this variation could be attributed to the amounts of Cu sequestered by both plants in the high Cu treatment in the initial experiment, which as previously mentioned is likely variable. Lastly, it is also important to consider that this experiment was conducted in a lab under controlled conditions that are not fully representative of natural aquatic ecosystems, where native microbial communities would be present. While microbes were certainly present within our mesocosms, we did not specifically manipulate microbe communities to include wetland sediments and their associated taxa. Microbes have been shown to mediate decomposition in another duckweed species, *L. gibba*, by increasing rates of decomposition threefold when compared to an axenic environment [58] and decomposition rates were shown to be high in *V. giganta* when left in an aquatic field site [59]. As a result, native microbe exclusion within the experimental design may not mimic natural decomposition processes and accurately depict degree of leaching across species. Further work that manipulates microbial density and diversity will provide additional insight into the leaching of Cu following phytoremediation efforts in natural ecosystems.

The decomposition of aquatic plant tissues may also be influenced by annual FTCs. Here, we found that artificial FTCs led to an increase in mortality rates for snails exposed to duckweed tissue from the high Cu treatment, but not the low Cu treatment. These results show that FTCs may stimulate breakdown of organic matter and expedite decomposition. This aligns with other studies that show decomposition of leaf tissue in both terrestrial and aquatic plants correlates with the frequency of freeze-thaw cycles [32,34]. Although the mechanism for the increased decomposition of plant tissue cannot be elucidated from this study, a potential mechanism of action is that freeze-thaw cycles cause physical weathering, breakdown, and erosion of plant tissue and consequently cause leaching [60]. In management considerations of *L. minor* as a phytoremediator, removal may be necessary before the cold season to ensure leaching does not occur back into the environment after sequestration. The effect of freeze-thaw cycles on decomposition have also been shown to be mediated through increased susceptibility to decomposers as an indirect effect of damage by freeze-thaw cycles on the structural integrity of litter and nutrient release [61]. Future research should account for the possibility of an interplay between decomposer activity and plant tissue damage during decomposition under freeze-thaw cycles.

## 5 Conclusions

Duckweed and tape grass have been shown to be effective phytoremediators in environments contaminated with chemical pollutants, including Cu. Our study provides insight on the phytoremediation capabilities of duckweed and tape grass, whether they leach Cu back into the environment after plant death, and the effect of freeze-thaw cycles on leaching potential. Our results suggest that at low Cu concentrations (i.e., Cu concentrations used for algicide purposes), duckweed is a more effective phytoremediator of Cu compared to tape grass. We found that at high Cu concentrations (i.e., Cu concentrations measured in runoff events), there is some evidence for phytoremediation, but decomposition of plant tissues used in phytoremediation efforts can leach harmful levels of Cu back into aquatic environments. Future research should vary environmental conditions, including utilizing finer substrates and integrating native microbial communities, to obtain study systems reflective of natural ecosystems.

## Author contributions

Conceptualization, G.M. and J.H.; methodology, B.E., G.M., G., V.W., D.A., A.B., B.B., A.D., K.H., L.K., M.M., E.M., A.W., Y.Z., J.G., D.C., and J.H.; formal analysis, J.H.; resources, G.M., J.G., D.C. and J.H.; writing—original draft preparation, B.E., G.M., G., V.W., D.A., A.B., B.B., A.D., K.H., L.K., M.M., E.M., A.W., Y.Z., J.G., D.C., and J.H.; writing—review and editing, B.E., G.M., G., V.W., D.A., A.B., B.B., A.D., K.H., L.K., M.M., E.M., A.W., Y.Z., J.G., D.C., and J.H.; supervision, G.M., and J.H.; project administration, G.M., and J.H.; funding acquisition, G.M., J.G., D.C. and J.H. All authors have read and agreed to the published version of the manuscript.

## Funding

Funding provided by 10.13039/100008451Binghamton University to G.M., J.G., D.C., J.H. and NSF CAREER award #2042970 to J.H.

## Declaration of competing interest

The authors have declared that no competing interests exist.

## Figures and Tables

**Table:** 

	Plant and copper treatments					
	Duckweed 0 ppm	Duckweed 1 ppm	Duckweed 10 ppm	Tape grass 0 ppm	Tape grass 1 ppm	Tape grass 10 ppm
**Experiment 1, time point 1**	0.079 (0.028)	0.20 (0.056)	0.39 (0.1)	0.09 (0.027)	0.15 (0.033)	0.24 (0.047)
**Experiment 1, time point 2**	0.023 (0.0081)	0.068 (0.011)	0.11 (0.021)	0.003 (0.0031)	0.056 (0.029)	0.11 (0.012)
**Experiment 1, time point 3**	0.031 (0.021)	0.11 (0.014)	0.12 (0.023)	0.019 (0.023)	0.045 (0.0035)	0.074 (0.007)
**Experiment 2, leachate solutions**	0.03	0.021	0.065	0.016	0.016	0.089
**Experiment 2, freeze/thaw leachate solutions**	0.07	0.028	0.055	NA	NA	NA

## Appendix 1

Copper concentrations (mean and standard deviation) from water samples used in Experiments 1 and 2 (N = 8 per plant/treatment combination). Time point 1 was immediately after Cu treatment, time point 2 was four weeks after treatment, and time point 3 was eight weeks after treatment. Standard deviations are not reported for Experiment 2 leachate solutions as there was no sample replication for analysis (i.e., a single leachate solution was generated for each treatment, and then subsampled to generate experimental units).

## References

[1] Driscoll D.A., Bland L.M., Bryan B.A., Newsome T.M., Nicholson E., Ritchie E.G., Doherty T.S. (2018). A biodiversity-crisis hierarchy to evaluate and refine conservation indicators. Nat. Ecol. Evol..

[2] Kummu M., de Moel H., Ward P.J., Varis O. (2011). How close do we live to water? A global analysis of population distance to freshwater bodies. PLoS One.

[3] Strayer D.L., Dudgeon D. (2010). Freshwater biodiversity conservation: recent progress and future challenges. JNBS.

[4] Green P.A., Vörösmarty C.J., Harrison I., Farrell T., Sáenz L., Fekete B.M. (2015). Freshwater ecosystem services supporting humans: pivoting from water crisis to water solutions. Global Environ. Change.

[5] Gleick P.H., Palaniappan M. (2010). Peak water limits to freshwater withdrawal and use. Proc. Natl. Acad. Sci. U. S. A..

[6] Havel J.E., Kovalenko K.E., Thomaz S.M., Amalfitano S., Kats L.B. (2015). Aquatic invasive species: challenges for the future. Hydrobiologia.

[7] Carpenter S., Stanley E., Zanden M.J. (2011). State of the world's freshwater ecosystems: physical, chemical, and biological changes. Annu. Rev. Environ. Resour..

[8] Ali H., Khan E., Ilahi I. (2019). Environmental chemistry and ecotoxicology of hazardous heavy metals: environmental persistence, toxicity, and bioaccumulation. J. Chem..

[9] Rehman M., Liu L., Wang Q., Saleem M.H., Bashir S., Ullah S., Peng D. (2019). Copper environmental toxicology, recent advances, and future outlook: a review. Environ. Sci. Pollut. Res..

[10] Wuana R.A., Okieimen F.E. (2011). Heavy metals in contaminated soils: a review of sources, chemistry, risks and best available strategies for remediation. ISRN Ecol..

[11] Mehta R., Templeton D.M., O’brien P.J. (2006). Mitochondrial involvement in genetically determined transition metal toxicity II. Copper toxicity. Chem. Biol. Interact..

[12] Gaetke L.M., Chow-Johnson H.S., Chow C.K. (2014). Copper: toxicological relevance and mechanisms. Arch. Toxicol..

[13] Taylor A.A., Tsuji J.S., Garry M.R., McArdle M.E., Goodfellow W.L., Adams W.J., Menzie C.A. (2020). Critical review of exposure and effects: implications for setting regulatory health criteria for ingested copper. Environ. Manag..

[14] Agency for Toxic Substances and Disease Registry (ATSDR) (2004).

[15] Environmental Protection Agency (2019).

[16] Kiaune L., Singhasemanon N. (2011). Pesticidal copper (I) oxide: environmental fate and aquatic toxicity. Rev. Environ. Contam. Toxicol..

[17] Hall J., Scott M.C., Killen W.D. (1998). Ecological risk assessment of copper and cadmium in surface waters of chesapeake bay watershed. Environ. Toxicol. Chem..

[18] de Oliveira-Filho E.C., Lopes R.M., Paumgartten F.J.R. (2004). Comparative study on the susceptibility of freshwater species to copper-based pesticides. Chemosphere.

[19] Lake-Thompson I., Hofmann R. (2019). Effectiveness of a copper based molluscicide for controlling Dreissena adults. Environ. Sci.: Water Res. Technol..

[20] Parra G., Jiménez-Melero R., Guerrero F. (2005). Agricultural impacts on mediterranean wetlands : the effect of pesticides on survival and hatching rates in copepods. Ann. Limnol. - Int. J. Lim..

[21] García-Muñoz E., Guerrero F., Parra G. (2009). Effects of copper sulfate on growth, development, and escape behavior in epidalea calamita embryos and larvae. Arch. Environ. Contam. Toxicol..

[22] Roussel H., Ten-Hage L., Joachim S., Le Cohu R., Gauthier L., Bonzom J.-M. (2007). A long-term copper exposure on freshwater ecosystem using lotic mesocosms: primary producer community responses. Aquat. Toxicol..

[23] Qasem N.A.A., Mohammed R.H., Lawal D.U. (2021). Removal of heavy metal ions from wastewater: a comprehensive and critical review. npj Clean Water.

[24] Fu F., Wang Q. (2011). Removal of heavy metal ions from wastewaters: a review. J. Environ. Manag..

[25] Ali H., Khan E., Sajad M.A. (2013). Phytoremediation of heavy metals—concepts and applications. Chemosphere.

[26] Ali S., Abbas Z., Rizwan M., Zaheer I.E., Yavaş İ., Ünay A., Abdel-Daim M.M., Bin-Jumah M., Hasanuzzaman M., Kalderis D. (2020). Application of floating aquatic plants in phytoremediation of heavy metals polluted water: a review. Sustainability.

[27] Burges A., Alkorta I., Epelde L., Garbisu C. (2018). From phytoremediation of soil contaminants to phytomanagement of ecosystem services in metal contaminated sites. Int. J. Phytoremediation.

[28] van der Ent A., Baker A.J.M., Reeves R.D., Pollard A.J., Schat H. (2013). Hyperaccumulators of metal and metalloid trace elements: facts and fiction. Plant Soil.

[29] Rai P.K. (2008). Heavy metal pollution in aquatic ecosystems and its phytoremediation using wetland plants: an ecosustainable approach. Int. J. Phytoremediation.

[30] Anand S., Bharti S.K., Kumar S., Barman S.C., Kumar N., Arora N.K., Kumar N. (2019). Phyto and Rhizo Remediation.

[31] Rai P.K. (2009). Heavy metal phytoremediation from aquatic ecosystems with special reference to macrophytes. Crit. Rev. Environ. Sci. Technol..

[32] Zhu J., Yang W., He X. (2013). Temporal dynamics of abiotic and biotic factors on leaf litter of three plant species in relation to decomposition rate along a subalpine elevation gradient. PLoS One.

[33] Wu F., Yang W., Zhang J., Deng R. Litter (2010). Decomposition in two subalpine forests during the freeze–thaw season. Acta Oecol..

[34] Whitfield C.J., Casson N.J., North R.L., Venkiteswaran J.J., Ahmed O., Leathers J., Nugent K.J., Prentice T., Baulch H.M. (2019). The effect of freeze-thaw cycles on phosphorus release from riparian macrophytes in cold regions. Can. Water Resour. J./Revue can. ressour. hydriques.

[35] Zayed A., Gowthaman S., Terry N. (1998). Phytoaccumulation of trace elements by wetland plants: I. Duckweed. J. Environ. Qual..

[36] Parra L.-M.M., Torres G., Arenas A.D., Sánchez E., Rodríguez K., Ahmad P., Prasad M.N.V. (2012). Abiotic Stress Responses in Plants: Metabolism, Productivity and Sustainability.

[37] Bokhari S.H., Ahmad I., Mahmood-Ul-Hassan M., Mohammad A. (2016). Phytoremediation potential of Lemna minor L. For heavy metals. Int. J. Phytoremediation.

[38] Ekperusi A.O., Sikoki F.D., Nwachukwu E.O. (2019). Application of common duckweed (Lemna minor) in phytoremediation of chemicals in the environment: state and future perspective. Chemosphere.

[39] St-Cyr L., Campbell P.G. (2000). Bioavailability of sediment-bound metals for Vallisneria americana michx, a submerged aquatic plant, in the st. Lawrence river. Can. J. Fish. Aquat. Sci..

[40] USDA, NRCS (2022). http://plants.usda.gov.

[41] Biernacki M., Lovett-Doust J., Lovett-Doust L. (1997). Laboratory assay of sediment phytotoxicity using the macrophyte Vallisneria americana. Environ. Toxicol. Chem..

[42] Wang Q., Li Z., Cheng S., Wu Z. (2010). Influence of humic acids on the accumulation of copper and cadmium in Vallisneria spiralis L. From sediment. Environ. Earth Sci..

[43] Rai P.K., Tripathi B.D. (2009). Comparative assessment of Azolla pinnata and Vallisneria spiralis in Hg removal from G.B. Pant sagar of singrauli industrial region, India. Environ. Monit. Assess..

[44] Liu H., Meng F., Tong Y., Chi J. (2014). Effect of plant density on phytoremediation of polycyclic aromatic hydrocarbons contaminated sediments with Vallisneria spiralis. Ecol. Eng..

[45] He Y., Chi J. (2019). Pilot-scale demonstration of phytoremediation of PAH-contaminated sediments by hydrilla verticillata and Vallisneria spiralis. Environ. Technol..

[46] Dillon R.T., Wethington A.R., Rhett J.M., Smith T.P. (2002). Populations of the European freshwater pulmonate Physa acuta are not reproductively isolated from American Physa heterostropha or Physa integra. Invertebr. Biol..

[47] Kefford B.J., Nugegoda D. (2005). No evidence for a critical salinity threshold for growth and reproduction in the freshwater snail Physa acuta. Environ. Pollut..

[48] Gao L., Doan H., Nidumolu B., Kumar A., Gonzago D. (2017). Effects of copper on the survival, hatching, and reproduction of a pulmonate snail (Physa acuta). Chemosphere.

[49] Mal T.K., Adorjan P., Corbett A.L. (2002). Effect of copper on growth of an aquatic macrophyte, elodea canadensis. Environ. Pollut..

[50] Ater M., Ait Ali N., Kasmi H. (2006). Tolerance and accumulation of copper and chromium in two duckweed species: Lemna minor L. And Lemna gibba L. Rev. Sci. Eau.

[51] Obermeier M., Schröder C.A., Helmreich B., Schröder P. (2015). The enzymatic and antioxidative stress response of Lemna minor to copper and a chloroacetamide herbicide. Environ. Sci. Pollut. Res. Int..

[52] Bai L., Liu X.-L., Hu J., Li J., Wang Z.-L., Han G., Li S.-L., Liu C.-Q. (2018). Heavy metal accumulation in common aquatic plants in rivers and lakes in the taihu basin. Int. J. Environ. Res. Publ. Health.

[53] Haile T.M., Fuerhacker M. (2018). Simultaneous adsorption of heavy metals from roadway stormwater runoff using different filter media in column studies. Water.

[54] Liu Y., Xu H., Yu C., Zhou G. (2021). Multifaceted roles of duckweed in aquatic phytoremediation and bioproducts synthesis. GCB Bioenergy.

[55] Dhabab J.M. (2011). Removal of some heavy metal ions from their aqueous solutions by duckweed. JTEHS.

[56] Li X., Cui B., Yang Q., Tian H., Lan Y., Wang T., Han Z. (2012). Detritus quality controls macrophyte decomposition under different nutrient concentrations in a eutrophic shallow lake, North China. PLoS One.

[57] Ping Y., Pan X., Cui L., Li W., Lei Y., Zhou J., Wei J. (2017). Effects of plant growth form and water substrates on the decomposition of submerged litter: evidence of constructed wetland plants in a greenhouse experiment. Water.

[58] Szabó S., Braun M., Nagy P., Balázsy S., Reisinger O. (2000). Decomposition of duckweed (Lemna gibba) under axenic and microbial [-2pt] conditions: flux of nutrients between litter water and sediment, the impact of leaching and microbial degradation. Hydrobiologia.

[59] Shilla D., Asaeda T., Fujino T., Sanderson B. (2006). Decomposition of dominant submerged macrophytes: implications for nutrient release in myall lake, NSW, Australia. Wetl. Ecol. Manag..

[60] LiPing J., Kai Y., YuLian Y., QingGui W. (2016). Leaching and freeze-thaw events contribute to litter decomposition - a review. Sains Malays..

[61] Taylor B.R., Parkinson D. (1988). Does repeated freezing and thawing accelerate decay of leaf litter?. Soil Biol. Biochem..

